# Public Health in the Headlines: A Study of Media Behavior on Discourses on Vaccination During COVID-19

**DOI:** 10.3390/vaccines13090937

**Published:** 2025-09-02

**Authors:** Carolina Jann Scalfoni, Edson Theodoro dos Santos Neto, Tatiana Breder Emerich

**Affiliations:** Graduate Program in Collective Health, Federal University of Espírito Santo, Vitória 29043-900, Brazil; edson.t.santo@ufes.br (E.T.d.S.N.); tatianaemerich@gmail.com (T.B.E.)

**Keywords:** COVID-19, vaccination, media coverage, Brazil, pandemic

## Abstract

Background/Objectives: The COVID-19 pandemic was characterized by the rapid transmission of the virus and a global race for vaccines, with vaccines such as AstraZeneca, CoronaVac, Pfizer, and Janssen arriving in Brazil in 2020. Concurrently, an infodemic of information, driven by the media and social media, highlighted the importance of health communication. This study examines how online newspapers in a Brazilian state disseminated information about vaccination and its relationship with vaccine adherence among the population. Methods: Quantitative research, in which a total of 5308 journalistic articles were verified, using two databases, one for the publication of journalistic articles and the other for vaccinations in the state, which applied 9,577,567 doses in the period. Results: The analyses demonstrated a positive correlation between the number of publications of articles and the number of applications of vaccines (rho = 0.407, *p*-value < 0.0005), revealing a relationship of both increase and decrease in the publication of newspaper articles and the application of vaccines in specific weeks during the analysis period. Vaccination data revealed low adherence to the booster dose by the population, with unequal values among the cities of the state. Conclusions: The study highlighted the potential importance of newspapers in disseminating information about vaccines during the pandemic, underscoring the need for regional health strategies to increase vaccination coverage.

## 1. Introduction

The COVID-19 pandemic, caused by the coronavirus, emerged at the end of 2019, in the city of Wuhan, China, through a mutation of a coronavirus, which caused a respiratory syndrome (SARS-CoV-2), and its beginning was characterized by a high rate of transmission of the disease between people [1]. In 2020, several vaccine companies had developed prototypes to begin manufacturing their vaccines. Some of these prototypes arrived in Brazil to initiate vaccination nationwide [2].

One of the characteristics of the new coronavirus pandemic was the widespread circulation of information about this new virus and the disease it caused among the population. The concept of an information infodemic was adapted and applied to the topic, highlighting the volume of information produced during the period, primarily through media, telecommunications, and information companies. Additionally, the impact on social networks was one of the most notable observations [3].

In this context, Communication and Health, which is part of a diverse field of education, information, and science [4], reflects the need for good communication and the seriousness of transmitting health information to the population, especially at a time of newness in the area of science, with the lack of knowledge about the virus. This role of the field aims to reach all audiences and is also essential for strengthening the ties between media institutions and the dissemination of science and health. In an atypical moment of the pandemic, it becomes a crucial source of information and of extreme importance for a country’s public health [5].

Given the relationship that the media has with health and the population as a vehicle for sharing news articles, it is necessary to understand how health news circulated during the pandemic period, what its content was, and how the information helped the population to stay informed in the face of the emergence of a new disease that affected everyone.

Therefore, this work aims to analyze the media information published in 21 newspapers in the state of Espírito Santo, Brazil, about vaccination during the pandemic and relate it to the application of doses in the population, to verify the relationship between media and vaccination coverage.

## 2. Materials and Methods

### 2.1. Study Design

This is a quantitative, documentary, descriptive, and exploratory research study, in which an analysis of journalistic articles from 21 news companies in a state in Brazil was conducted, covering the period of the coronavirus pandemic and providing information on vaccines and vaccination in the state.

### 2.2. Research Setting

The present study analyzed data from a state in Brazil and its indices regarding journalistic articles and vaccination against COVID-19. According to the 2022 geographic census, produced by the Brazilian Institute of Geography and Statistics (IBGE), the state currently has 3,833,712 inhabitants, with a similar distribution by sex and age group, with the majority being adults. In terms of race and color, the population is predominantly brown, followed by white and black [6]. The state was chosen for its transparency in information on coronavirus data, being considered first place in data transparency in the country by the NGO Open Knowledge Brasil [7].

The study utilized various platforms and tools to collect data.

The COVID-19 Panel is an online tool created by the government of Espírito Santo to access information about the coronavirus and its impact on health indicators in the state. According to the COVID-19 Panel, the state had 1,384,793 confirmed cases of the disease, 15,214 deaths, and 9,577,567 doses administered as of April 2025. When compared to the most recent geographic census, it is evident that the confirmed cases account for nearly half of the state’s total population, indicating the pandemic’s significant impact on the state.

The SIGCOVID-19 tool was a fundamental part of the data collection in the research. The tool analyzes and monitors journalistic articles published by the state’s news companies about the COVID-19 pandemic, covering 21 electronic newspapers that take into account their regional representation and the newspapers’ audiences.

Research Electronic Data Capture REDCap (Vanderbilt University, Nashville, TN, USA) (https://redcap.ufes.br/surveys/?s=KFAMTTKLN9, accessed on 24 April 2025) is a free software that has tools to assist in data management. The platform has a specific form for SIGCOVID-19, and the researchers utilized the platform to store information collected from journalistic news, ensuring a more thorough analysis of the data with greater quality and efficiency.

### 2.3. Data Analysis

The period chosen for data collection was from 29 December 2019 to 6 May 2023, encompassing the period from the emergence of the first cases of the disease, starting in the first epidemiological week (Epi Week) of the year, until the end of the pandemic as declared by the World Health Organization. The syntax and Boolean operators chosen were “AND vaccine OR coronavirus OR covid”. In SIGCOVID-19, using the syntax and Boolean operators described, a total of 15,231 news articles were identified, categorized by year as follows: 2019—0 articles, 2020—1596 articles, 2021—12,180 articles, 2022—1447 articles, and 2023—8 articles.

To be included in RedCap, the news was read item by item, one at a time. The news passed through an exclusion criterion with three different aspects: (1) repeated news, in the same or different newspapers; (2) news classified as off-topic; (3) news where COVID-19 vaccination appeared only as a link. After the exclusion process, the researchers categorized the news articles as follows: 2019—0 articles, 2020—716 articles, 2021—4103 articles, 2022—487 articles, and 2023—2 articles. The research continued with 5308 news articles to analyze in the study, excluding 9923 additional news articles. After this step, the data registered in RedCap was downloaded into a CSV spreadsheet and transformed into a Microsoft Office Excel 2019 model, where we manually reviewed the data. The next step was to analyze data from the Vaccination Panel—Dose Application, which involved downloading and studying the available spreadsheet using SPSS Statistics 21.0 for Windows software (International Business Machines Corporation, NY, USA). We categorize the data into predetermined categories, as presented in Table 1.

To present the data, absolute and relative frequency tables were created for both banks, providing indicators of the information collected. Since the samples did not follow a normal distribution, we employed Spearman’s statistical analysis to examine the correlation between the two quantitative variables.

## 3. Results

A total of 5308 journalistic articles about COVID-19 vaccines were located from 29 December 2019 to 6 May 2023 in the 21 electronic newspapers included in the SIGCOVID-19 platform. Table 1 illustrates the distribution of this data across the various electronic newspapers, using the same categories as the RedCap form used to collect the information from the news articles.

Among the twenty-one newspapers analyzed, three stood out: A Gazeta, Site Barra, and ES Hoje, which together accounted for 2585 journalistic articles, or 48.7% of the total publications. On the other hand, some newspapers had lower rates, including Tribuna Online, ES Acontece, and Site de Linhares, which collectively published only 61 journalistic articles, accounting for a relative frequency of 1.1%.

Examining the region’s coverage, the Grande Vitória region, which is home to the state capital, has the largest number of articles on vaccines, accounting for 46.8% of the total number of articles, followed by the South region, which also has a considerable number of publications.

During the pandemic period from 2020 to 2023, we found variations in the quantity of publications. The year 2021 has the highest number, with 77.4% of total publications. In contrast, the year with the lowest number of articles was 2023, with only two news items. These variations in the quantity of publications illustrate different phases of the pandemic, as the years between the pandemic show the variability of the numbers of deaths, virus infections, and the initiation of vaccinations in the state, among other factors.

In the news analyzed, it is evident that the information space dominates most articles, followed by the opinion space. It is interesting to examine the different appearances that this space presents. In the table, the column format stands out compared to the others, as it represents approximately 60% of the total.

Visual resources are present in both printed and electronic newspapers. The table shows the presence of visual elements in most publications, with a total of 88.5% of articles having at least one graphic element. Photography, illustrations, and audio are the main features found. On the other hand, there is a low number of components, such as infographics, tables, or boxes.

The cited sources are also part of the journalistic articles. The emphasis is mainly on official government sources, organizations, societies, and agencies. However, some other sources did not receive as much attention, for example: citizens, health professionals, professional class councils, labor unions, researchers, and research in general.

The sections are characterized according to the preference of each newspaper, which is why the study observes the presence of diverse sections. The table displays some of the most prominent categories, including “Health”, “Brazil”, and “General”, which collectively account for 54.4%, or more than half, of the publications. In addition to the editorial offices, journalistic agencies are also present. These are companies that provide content for newspapers to use in their news articles. The companies create the news articles and make them available to the public through newspapers and other media outlets. During data collection, a minority of news reports utilize information from a specific agency, with only 8.2% using this content. There is a notable emphasis on Agency Brasil and Estadão Conteúdo.

**Figure 1:**
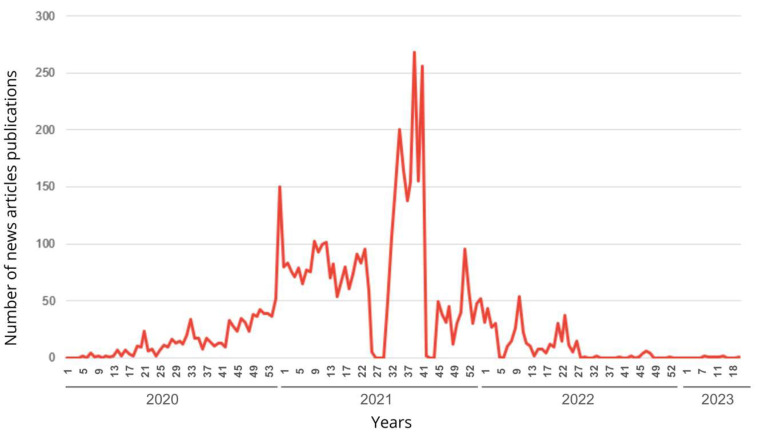
Peaks of publication of news articles about the vaccination in the state of Espírito Santo in electronic newspapers over the four years of the COVID-19 pandemic. Espírito Santo, Brazil, 2020 to 2023.

In addition to the results in Table 1, the graphic visualization of material publications over the four-year study period is also interesting. Figure 1 shows the publication curves found during the study period.

When analyzing the graph, 2020 begins with a low number of publications. As the year passes, a gradual increase in publications appears, indicating that the amount of news published increases by the Epi Weeks and the progression of the disease. The year 2021 saw a surge in publications, with rates significantly higher than those of the previous year. 2021 presents the prominent peaks on the graph, in Epi Week 33, 38, and 40, with Epi Week 38 being the one with the highest number of publications. After these weeks, it is already possible to identify a decrease in the number of publications, and 2022 represents this trend well. The year had an increase in publications in Epi Week 25. However, from the 29th onwards, a drop in quantity began that continued until 2023. This year, still considered to be in a pandemic state, had only two articles published until June, when the WHO declared the end of the global pandemic state.

Through the COVID-19 Panel in the state of Espírito Santo, it was possible to obtain information on COVID-19 vaccination throughout the state. Thus, using information obtained from the panel, Table 2 was created, specifying information about immunization in the state.

Among the four vaccine industries present in the state, Pfizer had the most significant number of applications, accounting for 40% of the total, followed by Oxford–AstraZeneca and CoronaVac/Butantan.

Some sociodemographic data were collected from the COVID-19 Panel, including sex and race/color. The sex with the highest prevalence of vaccinated people is female. Regarding race/color, the white, brown, and yellow populations obtained the highest percentages; however, it is possible to observe the presence of the subtopic “without information” with significant numbers in the two indices analyzed, leading to a decrease in the precision of these collected data.

The government of Espírito Santo established the COVID-19 Panel and presented its metrics about its population. As a result, it was possible to identify that the majority of those vaccinated are citizens of Espírito Santo. At the same time, the other 4% that belong to other states indicate the population that has been immunized in Espírito Santo but resides in different states in Brazil. The state government created groups based on specific categories. Specifically, 15.8% of the vaccinated population was elderly, and 9.5% had comorbidities, a population prioritized at the beginning of the vaccination rollout. Health workers account for only 3.1% of the data provided; however, with 69% of the data lacking information, these numbers may be higher.

**Figure 2:**
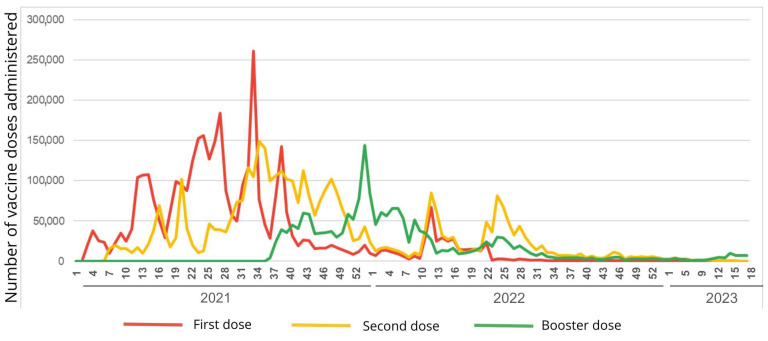
Vaccination peaks about the first, second, and booster doses in the state of Espírito Santo through the years (years of the vaccination’s initiation in Brazil, through to the end of the pandemic worldwide). Espírito Santo, Brazil, 2021 to 2023.

Figure 2 shows the three years of vaccine application until the end of the pandemic state, relating the amount of application to the Epi Week and displaying curves for each dose.

In 2021, a significant vaccination peak occurred, primarily in Epi Weeks 26, 33, and 38, with Epi Week 33 experiencing over 250,000 dose applications. The year contains a gradual increase in the number of vaccinations for the first and second doses. In contrast, the booster dose begins after Epi Week 34, consistent with the start of vaccine application.

The year 2022 begins with a drop in the number of vaccinations. This year, the booster dose takes a larger share compared to the first two doses in the first Epi Week of the year. It also experiences some peaks throughout the year, especially with the second dose, as observed in Epi Week 25. At the end of the year, there is a decrease in the three doses, with the lowest numbers since the beginning of vaccination.

Finally, in the last year analyzed, which spans the data collected up to the 18th Epi Week (the end of the pandemic), there was a drop in the number of vaccination doses administered in the state. When analyzing the rates of the first doses, they are almost zero. However, this metric reveals that the majority of the population had already received at least the first and second doses by the end of 2023, as shown in Table 2. The dose that stands out most in terms of the number of applications is the booster dose, although its rate remains low compared to previous years.

Utilizing the Spearman correlation, we found that the two variables analyzed showed a moderate positive correlation throughout the analyzed period. The year 2020 was excluded from the analysis, as the vaccination variable only began in the following year.

## 4. Discussion

### 4.1. The Findings in the Journalist Articles

The variable quantities of journalistic articles and number of COVID-19 vaccinations showed significant increases in specific Epi Weeks, among them the most related are Epi Week 33 and 38 of 2021, covering the first dose of vaccination, and Epi Week 25 of 2022, related to the second dose of vaccination for COVID-19, comprising peaks in both vaccination and publication of journalistic materials in both years. Furthermore, the number of articles published in the years following the pandemic is also related to the beginning of vaccine application. Since 2021, when vaccination began in the country, there has been a significant increase in SIGCOVID-19 publications, accounting for 77.4% of the total. These findings are related to the study by Neves and Massarani [8], which, when analyzing two national newspapers between 2019 and 2020, observed a significant increase in journalistic coverage of the topic in question. This upward trend, especially from December 2020, is also evident in Figure 1 of the present study. This increase in the publication of news articles continued until the end of 2021 in the case of the state of Espírito Santo.

**Figure 3:**
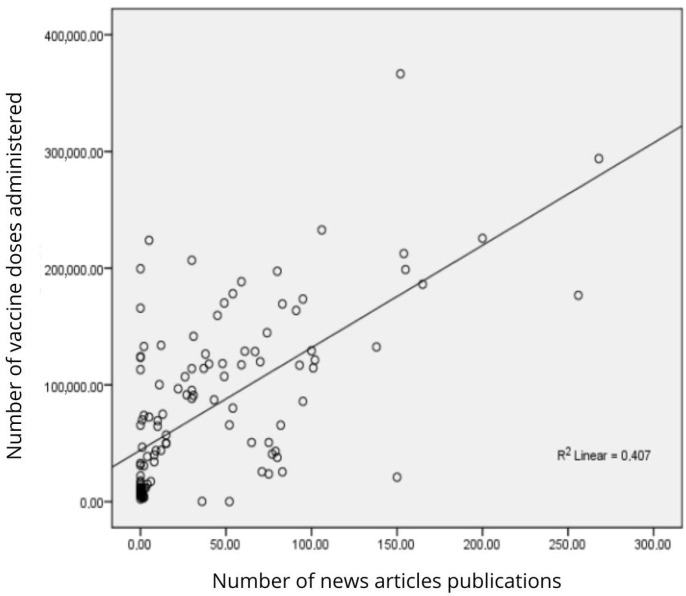
Analysis of the dispersion between the number of doses applied and publication of journalistic articles from 2021 to 2023, ES, Brazil.

Figure 3 shows the dispersion of dose and material values. When carrying out the analysis of 2021 and 2022 separately, 2021 presents a correlation gap between the indices; even though this is the year that presents the most significant quantity of them, the value found is 0.218, *p*-value > 5%, considered a negligible value, indicating that there is no correlation between the variables [9]. However, the year 2022 presents a strong positive correlation with a value of 0.805, *p*-value < 5%. This strong correlation is related to the decrease in materials and vaccinations in the state, which shows a strong correlation; however, it is associated with a reduction in variables throughout the study. So, for the entire period of analysis, from 2021 to 2023, it is possible to observe a moderate general correlation with 2 rho = 0.632; *p*-value < 0.001.

We can view this case as an example of the studies by Barrowman [10], which says that a positive correlation can accompany a negative causal relationship, as seen in our example, where the decrease in the number of news articles published over the years is associated. In Brazil, a large part of the population is informed through the media [11]. Analyzing the relationship between the population and the ways of information, it is possible to identify the role of the media in addressing current issues; as Cervi and Hedler [11] present in their studies, the most prevalent themes tend to reflect what is present in the daily lives of readers. Analyzing this fact, it becomes essential to recognize the correlation in our study and how the journals follow the distribution of news publications during the pandemic years.

In addition to quantitative analyses, it is essential to understand the behavior of media outlets and their production and/or dissemination of news articles. In a study conducted by Dos Santos Mota et al. [12] on the occupation of space in newspapers regarding vaccines and vaccination, the authors found that during the first weeks of the pandemic, informational news predominated. Over time, a trend toward service journalism was observed. About the news analyzed in Espírito Santo, most of the news articles are characterized as informative genre; however, unlike Dos Santos Mota et al. [12], in our study, the opinion genre occupied the second prominent place and not the service one as the other research cited, even though it has both, a small amount of news compared to the informative space.

Journalistic articles utilize visual resources to enhance the public’s understanding of the textual content. Therefore, their presence in the articles must be analyzed in terms of the target reading public and how they will be distributed [13]. The visual resource most found in the analysis of newspapers was photography, followed, in a significantly lower number, by illustrations. More than 80% of the articles contained editing elements in their content, with a notable presence in journalistic productions [12]. However, a counterpoint to analyze is the relevance of the images used. The use of generic photos in the preparation of a journalistic article can introduce ambiguity in some instances and not provide significant assistance when reading the text [14]. In this case, it is necessary to analyze whether the use of images, employed generically and repetitively, is the most effective editing mechanism to aid reading.

The COVID-19 pandemic led to an increase in the consumption of information and newspapers as reliable sources of information [15]. With a total of 92% of adults seeking information in the United States [15] and a 17% increase in television networks in Brazil [16], newspapers have become even more relevant. In this way, sources prove to be providers of helpful information for journalism [17]. Official sources gained greater prominence in the research carried out, being present in 74.1% of all articles in the study; these sources included government bodies in three instances, or their representatives. This large number of official sources brought by journalists is related to historical factors in the country, such as the military dictatorship and personal interests that involve both areas [17]. Despite the historical burden of official sources, a discrepancy is observed where study findings differ from those in other research, highlighting the relevance of professionals and portraying them as the primary sources used in journalistic articles, as found in the studies by Midões and Martins [18] and Lopes et al. [19].

### 4.2. The Information Contained in the COVID-19 Panel—Espírito Santo

The COVID-19 Panel, created by the state health department, collected information from the vaccinated population of Espírito Santo. Some of the information used in this study included the manufacturer of the vaccines, the doses applied, sex, race/color, the UF of the patients, and a small categorization of the group to which those vaccinated belonged.

The quality of data from information systems is crucial for an accurate assessment of a state’s health indicators [20]. Therefore, before analyzing the data, it is essential to note the topic “Without Information” that was found in the sample collected. A possible measure for quality analysis is to observe the percentage that this data occupies in the tables. A study conducted with the COVID-19 Panel evaluated data from children, adolescents, and young people and described the quality of the data as poor to fair in various variables, due to the high percentages of incomplete information [21]. In the present study, high percentages are also observed, as some metrics have a percentage of up to 69% for the data. This high percentage can limit the findings, since poor data quality can lead to false interpretations and generalizability, as described by the author.

Looking at the data collected, about the number of doses applied, we have data on the first and second doses that are very similar; however, when analyzing the applications of the booster doses, a drop of almost 50% in the quantity compared to the previous ones is notable, showing that a large part of the Espírito Santo population did not take the booster dose against the coronavirus. This finding is related to the study by Da Costa Borges and Dos Santos [22], which shows a tendency towards reduced vaccination with each subsequent dose.

The panel also provides information for each city individually. The vaccination coverage for the first and second doses exceeded 90% in most cities, with only six cities reporting percentages below this threshold. There was diversification between the cities in terms of coverage for the first and second doses. For the complete vaccination schedule, these percentages experienced significant drops; only five cities stood out, with percentages greater than 70%, with a particular emphasis on the capital, which has the highest rate at 82.03%. On the other hand, we have cities with very low rates, all with percentages below 40%, and a rural town with the lowest rate, with 28.45% of the population vaccinated with the full schedule. The lack of a complete vaccination schedule is also observed in other reports [23,24].

Population metrics are highlighted in the COVID-19 Panel. Notably, the female population, as well as the white and brown populations, are the most vaccinated in the state, with the highest vaccination numbers. Studies found values about sex, with 54% of doses applied to women in an analysis carried out with almost 9 million applications. Similar values are found in the present study, with 47.2% for women and a similar number of applications [22]. The authors associate this finding with the historical factor of greater concern for health among women.

When analyzing the race/color variable, it is possible to observe the presence of the “Without Information” category, which accounts for 22% of the total amount of information. This lack of information on such an essential variable for understanding the social situation is in all states of the country, with variations ranging from 8.72% to 39.41% [25]. This incompleteness, as the authors note, renders it impossible to analyze the sample with quality [21] and, consequently, to accurately verify the possible inequality found about race/color regarding COVID-19 vaccination, both in the state of Espírito Santo and nationwide [25].

The same lack of information is also found in the “Vaccinated Category”, which accounts for 69% of the total. The presence of specific categories consistent with the priority population at the beginning of vaccination includes individuals with comorbidities, healthcare workers, and older people. However, due to the large number of unrecorded data, it is not possible to carry out a relevant comparative analysis. The correct recording of this data is “essential for developing prevention, monitoring, policy formulation and resource allocation strategies” in the health area [26].

### 4.3. The Peak in the Publication of Journalistic Articles and Vaccination

The most prominent peaks in Figure 1, about the publication of articles, are observed in 2021, particularly between Epi Week 33 and 41. These weeks cover the months of August to October of the same year. When analyzing the peaks of vaccination, it is possible to identify significant similarities. The most prominent peaks in Figure 2, related to vaccination doses, are also evident in 2021, particularly in Week 33, when the first dose of the vaccine was administered, with more than 261,000 doses administered in just one Epi Week. In August 2021, especially during Epi Week 33, there were the highest numbers of published journalistic articles and administered vaccine doses.

It is known that the COVID-19 pandemic occurred through waves of disease and their variants [27]. Vaccination against the disease in Brazil began in January 2021 [28] and in the state of Espírito Santo in May of the same year [29], with a gradual increase in the target audience [28]. A study examining the different phases of the pandemic and their impacts on the population reveals that the period from August to October 2021 marked a milestone in vaccination speed, coinciding with a decrease in cases, deaths, and test positivity [30]. The study used “*Fiocruz’s MonitoraCovid-19*” system to collect this data across the entire Brazilian territory.

## 5. Conclusions

The results of the study demonstrate a moderate positive correlation between the publication of journalistic articles about vaccines and the administration of doses in the state of Espírito Santo. The highest publication peaks are observed in 2021, accounting for 77.4% of the total, particularly during Epi Weeks 33 to 38. This year, vaccination began in Brazil and Espírito Santo. Thus, there was greater journalistic coverage of COVID-19 vaccination in the year when the population started to be immunized. In contrast, there is a decrease in both study variables in the years 2022 and 2023, despite the pandemic still being ongoing, with a sharp decline in the production of journalistic content and a gradual decrease in vaccination rates.

The prevalence of informative materials in newspapers reflects a commitment to delivering high-quality information to the population of Espírito Santo. However, it is necessary to observe the prevalence of generic images in productions, which can hinder a greater understanding of the reading. When examining the quality of the data in the categories of the COVID-19 Panel—Espírito Santo, a high percentage of incomplete information is observed, which corroborates a lower capacity for inferences from the sample to the state’s population. An improvement in this collection is essential to help health demonstrators.

The study highlights disparities in journalistic coverage and the distribution of vaccine doses between different cities in the state of Espírito Santo, particularly regarding the booster dose of vaccination. A rural town in the state has the lowest rate among all. This analysis highlights the need for more regionally tailored health strategies in the state, particularly for cities with alarming data.

Despite the relevance of the study findings, which demonstrate a positive correlation between the analysis variables, it is essential to note that the study focuses on newspapers in the state of Espírito Santo, highlighting specific characteristics of the state and its population. It is interesting for future analyses to explore other communication strategies that promote greater adherence to booster doses of the vaccine, in addition to regional analyses in terms of inequality in journalistic articles and the application of vaccines with the first, second, and booster doses, in addition to possible analyses with other states in the country, so that comparisons can be made.

## Figures and Tables

**Table 1:** Newspaper articles, categorized by their structure and content, according to their specifications in the RedCap form. Percentages of each topic are shown in relation to the total number in each category. Espírito Santo, Brazil, December 2019 to May 2023.

Electronic Newspaper *N* = 5308	A Gazeta	957 (18.0%)
	Folha Vitória	281 (5.3%)
	ES Hoje	727 (13.7%)
	A Tribuna	32 (0.6%)
	Portal 27	87 (1.6%)
	Folha Online Es	131 (2.5%)
	Século Diário	250 (4.7%)
	Montanhas Capixabas	173 (3.3%)
	Notícia Capixaba	100 (1.9%)
	Aqui notícias	282 (5.3%)
	Jornal Fato	695 (13.1%)
	Folha Espírito Santo	59 (1.1%)
	Portal Maratimba	282 (5.3%)
	Em Dia ES	115 (2.2%)
	Site Barra	901 (17.0%)
	Rede Notícia ES	58 (1.1%)
	ES Acontece	1 (<0.1%)
	Site de Linhares	28 (0.5%)
	Eu vi em Linhares	52 (1.0%)
	ES 24 horas	97 (1.8%)
Coverage Region of the Journal *N* = 5308	Grande Vitória	2484 (46.8%)
	Serrana	285 (5.4)
	South	1291 (24.3)
	North	128 (2.4%)
	Northwest	941 (17.7%)
	North East	3 (0.1%)
	Rio Doce	176 (3.3%)
Year of Publication *N* = 5308	2020	712 (13.4%)
	2021	4107 (77.4%)
	2022	487 (9.2%)
	2023	2 (<0.1%)
Occupied space in the newspaper *N* = 5308	Informative	5054 (95.2%)
	Service	91 (1.7%)
	Publicities	2 (<0.1%)
	Opinion	161 (3.0%)
Sections of opinion articles *N* = 161	Cartoon	1 (0.7%)
	Author’s letters	5 (3.1%)
	Editorials	21 (13.0%)
	Article	38 (23.6%)
	Columns	96 (59.6%)
Visual resources in the articles *N* = 5308	Photography	4405 (83.0%)
	Infographic	77 (1.5%)
	Table/box	70 (1.3%)
	Illustration	276 (5.2%)
	Graphic	160 (3.0%)
	Video	160 (3.0%)
	Audio	182 (3.4%)
Cited sources *N* = 5308	Health professionals	472 (8.9%)
	Official (government)	3934 (74.1%)
	Citizens	374 (7.0%)
	NGO’s/Organizations/Societies/Agencies	844 (15.9%)
	Professional class council/Labor union	120 (2.3%)
	Research/researchers	480 (9.0%)
	Others	1005 (18.9%)
Sections *N* = 5308	Article	34 (0.6%)
	Brazil	811 (15.3%)
	Cities	174 (3.3%)
	Column	69 (1.1%)
	Coronavirus	112 (2.1%)
	Daily	466 (8.6%)
	Culture	19 (0.3%)
	Highlights	344 (6.4%)
	Economy	47 (0.8%)
	Education	57 (2.4%)
	Specials	8 (0.1%)
	Sports	18 (0.3%)
	State	214 (3.9%)
	Celebrities	16 (0.3%)
	General	717 (13.4%)
	Immunization	2 (<0.1%)
	Justice	9 (0.2%)
	World	115 (2.1%)
	News	371 (6.9%)
	Opinion	25 (0.5%)
	Police	8 (0.1%)
	Politics	201 (3.8%)
	Region	39 (0.7%)
	Health	1372 (25.7%)
	Others	60 (1.1%)
Agencies *N* = 5308	Agency Brasil	195 (3.7%)
	Agency Estado	38 (0.7%)
	Agency Folhapress	89 (1.8%)
	Agency Senado	9 (0.1%)
	Agency Câmara	5 (0.1%)
	Dino—Divulgador de Notícias	1 (<0.1%)
	Agency EFE	1 (<0.1%)
	Estadão Conteúdo	91 (1.7%)
	Fiocruz de Notícias	1 (<0.1%)
	Redação o Braço Sul	2 (<0.1%)
	Redação Multimídia ES Hoje	1 (<0.1%)
	Without agencies	4875 (91.8%)

**Table 2:** Classification of the population immunized in the state of Espírito Santo and their characteristics obtained from the COVID-19 Panel, created by the government of the state. The government established these categories, which we present in the table below for the period from 2021 to 2023 (the years of the vaccination’s initiation in Brazil, through to the end of the pandemic worldwide). Percentages for each specific topic are calculated based on the total number of immunizations in the state, marked as ‘total’ in each category. 95% CI refers to the confidence interval, a statistical concept used to indicate the precision of the calculated data values.

Categories		Number of Doses	Percentage (%)	95% CI
Vaccines	Coronavac/Butantan	1,781,558	18.6	18.58–18.63
	JANSSEN	721,214	7.5	7.51–7.55
	Oxford–AstraZeneca	3,122,023	32.6	32.57–32.63
	Pfizer	3,911,934	40.8	40.81–40.88
	Pfizer—Pediatric	40,838	0.4	0.42–0.43
	Total	9,577,567	100	
Doses of the vaccines	First dose	3,736,635	39	38.98–39.05
	Second dose	3,875,508	40.5	40.43–40.50
	Booster dose	1,965,424	20.5	20.50–20.55
	Total	9,577,567	100	
Sex	Female	4,525,319	47.2	47.22–47.28
	Male	3,795,490	39.6	39.60–39.66
	Without information	1,256,758	13.1	13.10–13.14
	Total	9,577,567	100	
Race/Color	Yellow	1,556,329	16.2	16.23–16.27
	White	2,961,269	30.9	30.89–30.95
	Indigenous	12,312	0.1	0.13–0.13
	Brown	2,391,293	25	24.94–25.00
	Black	501,030	5.2	5.22–5.25
	Without information	2,155,334	22.5	22.48–22.53
	Total	9,577,567	100	
State of the vaccinated	Another states	421,865	4.4	4.39–4.42
	Espírito Santo	9,155,702	95.6	95.58–95.61
	Total	9,577,567	100	
Categories of the vaccinated	Without information	6,606,787	69	68.95–69.01
	Teenagers	10	0	0–0
	People with comorbidities	906,578	9.5	9.45–9.48
	Security and Rescue Forces workers	45,136	0.5	0.47–0.48
	General population	27	0	0–0
	Elderly	1,504,841	15.8	15.69–15.74
	Education professionals	218,140	2.3	2.27–2.29
	Health professional	296,048	3.1	3.08–3.10
	Total	9,577,567	100	

## Data Availability

The COVID-19 Panel—Espírito Santo is a publicly available database https://coronavirus.es.gov.br/painel-covid-19-es (accessed on 20 August 2024) provided by the state government. The SIGCOVID-19 is a tool created by a group of researchers at the Federal University of Espírito Santo and is available on a specific computer at the university.

## Abbreviations

The following abbreviations are used in this manuscript:

Epi Week	Epidemiological Weekend
NGO	Non-governmental Organization
